# Anticipatory Cortical Activation Precedes Auditory Events in Sleeping Infants

**DOI:** 10.1371/journal.pone.0003912

**Published:** 2008-12-10

**Authors:** Tamami Nakano, Fumitaka Homae, Hama Watanabe, Gentaro Taga

**Affiliations:** 1 Graduate School of Education, University of Tokyo, Bunkyo-ku, Tokyo, Japan; 2 Japan Society for the Promotion of Science, Tokyo, Japan; 3 Core Research for Evolutional Science and Technology (CREST), Japan Science and Technology Agency, Saitama, Japan; Victoria University of Wellington, New Zealand

## Abstract

**Background:**

Behavioral studies have shown that infants can form associations between environmental events and produce anticipatory actions for the predictable event, but the neural mechanisms for the learning and anticipation of events in infants are not known. Recent neuroimaging studies revealed that the association cortices of infants show activation related to auditory-stimulus discrimination and novelty detection during sleep. In the present study, we expected that when an auditory cue (beeps) predicted an auditory event (a female voice), specific regions of the infant cortex would show anticipatory activation before the event onset even while sleeping.

**Methodology/Principal Findings:**

We examined the cortical activation of 3-month-old infants during delays between the cue and the event by using multi-channel near-infrared spectroscopy. To investigate spatiotemporal changes in cortical activation over the experimental session, we divided the session into two phases (early and late phase) and analyzed each phase separately. In the early phase, the frontal regions showed activation in response to the cue that was followed by the event compared with another cue that was not followed by any event. In the late phase, the temporoparietal region, in addition to the frontal region, showed prominent activation in response to the cue followed by the event. In contrast, when the cue was followed by an event and no-event in equal proportions, cortical activation in response to the cue was not observed in any phase.

**Conclusions:**

Sleeping 3-month-old infants showed anticipatory cortical activation in the temporoparietal and frontal regions only in response to the cue predicting the event, suggesting that infants can implicitly form associations between temporally separated events and generate the anticipatory activation before the predictable event. Furthermore, the different time evolution of activation in the temporoparietal and frontal regions suggests that these regions may be involved in different aspects of learning and predicting future events.

## Introduction

Our ability to learn regularities and associations in the external world is critical for adaptation, and this mechanism is potentially available early in life to guide perceptual development [1]. In fact, 3-month-old infants can learn the spatiotemporal pattern of visual presentations and make anticipatory eye movements in advance of stimulus onset [2]–[4]. Infants at 4 months of age showed longer fixation on a checkerboard pattern in the presence of an auditory cue that was forward-paired with a face stimulus compared with a cue that was random or backward-paired with a face stimulus [5]. These findings suggest that infants are able to form associations between environmental events and produce anticipatory responses. However, the neural mechanisms underlying these processes in infants remain unknown.

Previous neuroimaging studies with adults reported that the sensory and the association regions, including prefrontal cortex, showed cortical activation before stimulus onset when a preceding cue predicts the coming of the stimulus [6]–[11]. The top-down attentional control system involving the prefrontal cortex increases the activity in the sensory area that will respond to the upcoming stimulus [10], [12]. Despite the conventional view that the prefrontal cortex is too immature to function at a few months of age [13]–[15], recent neuroimaging studies with infants using near-infrared spectroscopy (NIRS) revealed that the prefrontal regions of 3-month-old infants were involved in stimulus discrimination [16] and in novelty detection [17]. Moreover, an event-related potential (ERP) study with 4-month-old infants reported a difference in presaccadic electrical activity between anticipatory and reactive saccades at the frontal sites [18]. Given these previous studies with infants, we expected that the prefrontal cortex and the sensory association regions of 3-month-old infants may exhibit anticipatory activation before the onset of a predictable event if they could form a temporal association between the events.

In the present study, 3-month-old infants were exposed to a series of auditory stimuli during sleep to examine their ability to associate temporally separated events. We prepared two different frequency beeps as auditory cues in the experimental group; one of them was followed by an attractive event of a female voice reading a book with a time delay, and the other was not followed by any event. For comparison to this group, we presented a single frequency beep as a cue that was followed by an event or no-event in equal proportions to the control group. We assumed that auditory information processing remained operative even when they were sleeping. This assumption is based on previous studies that showed that neural responses to speech sounds were observed during sleep in both infants [19]–[23] and adults [28], [29]. In addition, recent studies in adults demonstrated that some types of associative learning can implicitly occur without awareness [24], [25]. We further hypothesized that implicit processes related to learning of temporal associations between auditory events are active in infants during sleep. Thus we focused on the learning of temporal relations between auditory stimuli without interference by input from another modality such as vision.

To examine the cortical activation of the infants noninvasively and safely, we used a multi-channel NIRS system [16], [17], [20]–[22], [26]–[31]. Anticipatory cortical activation during a delay period between the cue and the event was assessed by detecting transient increases in oxy-Hb signals. The infants did not know the relation between the cue and the event at the initiation of the session, and the association between the cue and event was assumed to be established by exposure to the stimuli during the experimental session. Thus we investigated spatiotemporal changes in cortical activation over the experimental session.

## Methods

### Participants

Fifty-six full-term, healthy Japanese infants at 3 months of age (boys, *n* = 32; girls, *n* = 24; mean age, 110.6 days; range 99–123 days; SD 5.6) participated in the present study. Twenty-eight infants were allocated to the experimental group (boys, *n* = 16; girls, *n* = 12; mean age, 107.5 days; SD 5.4), and the other twenty-eight infants were allocated to the control group (boys, *n* = 16; girls, *n* = 12; mean age, 113.6 days; SD 4.0). An additional seventeen infants were tested but excluded from the analysis because of awakening during the experiment (*n* = 11), head movements producing large motion artifacts in the signals (*n* = 5), or failure in probe placement because of obstruction by hair (*n* = 1). The parents of the infants gave written informed consent before the initiation of experiments. These experiments were approved by the ethical committee of the Graduate School of Education (University of Tokyo).

### Stimuli

The cue was auditory beeps consisting of three repetitions of identical pure tones (400 Hz or 700 Hz, 250 ms duration) with a 250 ms inter-stimulus-interval (total duration, 1.25 s). A female voice reading the book *Winnie-the-Pooh* in Japanese was used as the auditory event. We prepared eight sentences, and the mean duration of each of them was 4.5 s.

### Experimental design

**Figure 1:**
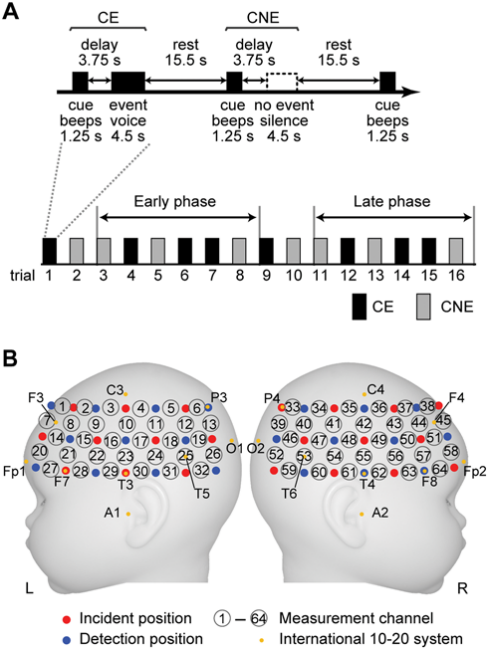
The experimental set-up for measurement. (A) An event-related paradigm used in the present study. The cues (beeps) were presented. One was followed by the auditory event (speech sounds) after a delay period (CE, Exp-CE and Cont-CE in the experimental and control groups, respectively), and the other was followed by no sounds (CNE, Exp-CNE and Cont-CNE in the experimental and control groups, respectively). The presentation order of the two conditions (CE and CNE) was fixed in one of two patterns in both groups. One is illustrated in the figure; the order of CE and CNE was reversed in the other pattern (counterbalanced across infants). The third to eighth trials (second to fourth trials of CE and CNE) were assigned to the early phase, and the eleventh to sixteenth trials (sixth to eighth trials of CE and CNE) were assigned to the late phase. (B) Arrangement of near-infrared spectroscopy (NIRS) probes. Measurement channels (circled numbers) were placed over bilateral frontal, temporal and temporoparietal regions of infants. Red-filled circles and blue-filled circles indicate incident positions and detection positions of NIRS, respectively. The distance between the closest incident and detection positions was approximately 2 cm. The yellow-filled circle indicates the positions of the international 10-20 system of electrode placement.

The experimental session consisted of two conditions: a cue was followed by an event with a delay (CE), and a cue was not followed by any event (CNE) as shown in Figure 1A. The delay period was set at 3.75 s (the time length from the cue onset to the event onset was 5 s). We expected that the infants were able to associate the cue with the event that was temporally separated. The delay period was comparable with that used in previous behavioral studies, in which 3-month-old infants were conditioned using a 5 s delay of reinforcement and 6-month-old infants performed the oculomotor response with delays of up to 5 s [32], [33]. Each presentation of an auditory event was followed by a rest period (15.5 s) for the cerebral blood flow induced by the event to return to baseline. Thus the trial length for each condition was 25 s. In the experimental group, one of two cues was assigned to CE (Exp-CE), and the other was assigned to CNE (Exp-CNE), counterbalanced across infants (Table 1). On the other hand, one of the two cues, counterbalanced across infants, was assigned to both CE and CNE in the control group (Cont-CE and Cont-CNE, respectively). We conducted 8 trials for each condition (total of 16 trials) with the expectation that only the infants of the experimental group under the CE condition would show anticipatory cortical activation during the delay period as the learning progressed. Although there was no overt response from the infants during sleep, the number of trials was determined based on the behavioral studies of conditioning in 4-month-old infants [5]. In both groups, the presentation order of the two conditions (CE and CNE) was fixed in either one of two patterns, counterbalanced across infants. One pattern was as illustrated in Figure 1A, and the other pattern was the reverse order of this.

**Table 1:** Frequency of beeps.

Group	Frequency of beeps		Number of infants
	CE^1^	CNE^2^	
Experimental group	400 Hz	700 Hz	n = 14
	700 Hz	400 Hz	n = 14
Control group	400 Hz	400 Hz	n = 14
	700 Hz	700 Hz	n = 14

1 A cue was followed by an event with a delay.

2 A cue was not followed by any event.

Infants were held by an experimenter who sat on a chair in front of the speaker in a sound-attenuated room. We tested during the daytime while infants were sleeping soundly and were almost motionless. The infants' sleeping state corresponded to quiet sleep (QS). QS is the dominant and stable state in daytime sleep of infants around 3 months old [34]. The status of infants was video recorded to identify the trials in which infants slept without moving their heads.

### NIRS recordings

We used an NIRS instrument (ETG-7000, Hitachi Medical Corporation, Tokyo, Japan). This instrument measured the time course of relative changes in the concentrations of oxyhemoglobin (oxy-Hb) and deoxyhemoglobin (deoxy-Hb) at multiple channels with 0.1 s time resolution by using two wavelengths of near infrared light (785 nm and 830 nm) and applying data analyses based on the modified Lambert–Beer law [35]. Because the precise optical path length was unknown, the unit of these values was a molar concentration multiplied by length (mM·mm). Detailed descriptions of the principles underlying NIRS have previously been published [35]–[38]. The NIRS instrument has been successfully used to investigate cortical activation in infants [17], [20], [22], [26], [28]–[31]. We placed two 3×7 arrays mounted on a flexible cap over the frontal, temporal, and temporoparietal regions of each hemisphere, as shown in Figure 1B. We set ten incident and eleven detection optical fibers on the left hemisphere and eleven incident and ten detection optical fibers on the right hemisphere. The distance between incident and detection positions was approximately 2 cm [39]. Each pair of adjacent incident and detection fibers defined a single measurement channel, which enabled us to simultaneously measure the time courses of oxy-Hb and deoxy-Hb signals at 64 measurement channels. The intensity of the near infrared light was set at 0.6 mW. The measured area in each hemisphere was correctly positioned by using the nasion, vertex, and pre-auricular points as skull landmarks in each infant. Measurement channel 10 (Ch10) and Ch23 on the left hemisphere and Ch42 and Ch55 on the right hemisphere were set on a coronal line from the pre-auricular point of one ear over the vertex to the opposite ear. The left Ch23 and right Ch55 were set 1 cm above the T3 and T4 positions, respectively, of the international 10-20 system of electrode placement. The left Ch7 and right Ch45 were set on the F3 and F4 positions, respectively. Studies in adults show that the T3 and T4 positions are projected around the middle and superior temporal gyri [40].

### Data analysis

We examined relative changes in oxy-Hb and deoxy-Hb signals, which estimate changes in regional cerebral blood oxygenation during brain activation. In each individual data set, baseline drifts were corrected by high-pass filtering, implemented using the fifth-order Butterworth filter with cut-off frequencies under 1/60 Hz. Then, we extracted trial data from the time-course data. Each trial data ranged from 1.0 s prior to the cue onset to 25.0 s after the cue onset. We adjusted the baseline by fitting the mean signal of the first 10 time points (1.0 s) to zero. By detecting rapid changes in the oxy-Hb signals, trial data with a low signal-to-noise ratio because of obstruction by hair and those that included movement artifacts were eliminated. We used infant data that contained a minimum of six good trial data for each condition. Following the screening process, we obtained data on 56 infants for further analysis. The mean number of trials was 7.4 in all conditions. By averaging the signal changes over trial data for each infant under each condition, we obtained the hemodynamic responses at each measurement channel. Then, we determined averaged time course among all measurement channels under each condition. In further analysis, we focused on oxy-Hb signals because their magnitude of relative signal changes was greater than those of deoxy-Hb signal changes [16], [17], [21], [26].

To examine spatiotemporal changes in cortical activation during the progress of the trials, we divided the experimental session into early and late phases (Figure 1A). Given that infants did not have any knowledge of the relation between the cue and the event in the first two trials (first trial of CE and CNE), we excluded them and divided other trials in a way that each phase contained the same number of trials in each condition. The third to eighth trials (second to fourth trials of CE and CNE) were assigned to the early phase, and the eleventh to sixteenth trials (sixth to eighth trials of CE and CNE) were assigned to the late phase. In each phase, we averaged signal changes over trial data for each subject under each condition at each measurement channel.

To identify the cortical regions that showed an increase in oxy-Hb signals during the delay period in the early and late phases, we calculated mean signal changes in the time range from 4.0 s to 5.0 s after the cue onset under each condition. We considered the individual data as random effects and performed *t* tests for each channel. First, a *t* test of the signal changes under each condition was performed against zero baselines (one tail). Then, we compared mean signal differences between conditions on a channel-by-channel basis in each group (paired *t* test, one tail). To identify the cortical regions that were activated in both early and late phases, we performed conjunction analysis of these *t* tests between phases [41]. Next, to examine the learning effect, we compared mean signal differences between the two phases under each condition (paired *t* test, one tail) and also compared the mean signal differences between the conditions in the early phase with those in the later phase (paired *t* test, one tail). We furthermore compared the mean signal differences between the conditions in the experimental group with those in the control group on a channel-by-channel basis (two-sample *t* test, one tail). We also examined cortical activation in response to the auditory events. We analyzed the maximum time points of the averaged time course in Exp-CE and Cont-CE (10.7 s and 10.5 s, respectively) and calculated mean signal changes in the time range from 9.5 s to 11.5 s in each phase for each infant. Then, the *t* tests for each channel were performed by considering the individual data as random effects (one-sample *t* test). To take into account multiple comparisons among 64 channels, we applied the False Discovery Rate correction with α = 0.05 in all analyses [42], [43].

## Results

**Figure 2:**
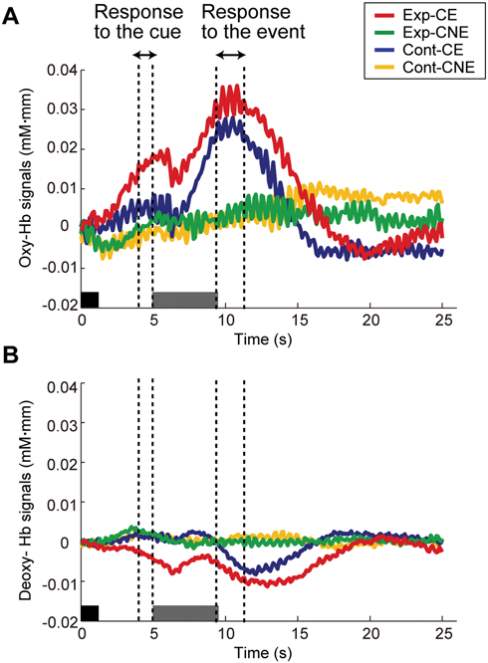
Mean time courses of hemodynamic response among all channels under each condition. While the oxyhemoglobin (oxy-Hb) signal changes (A) showed a positive response to the auditory events in Exp-CE (red line) and Cont-CE (blue line), the deoxyhemoglobin (deoxy-Hb) signal changes (B) showed a negative response. The dark gray bars and light gray bars indicate the cue period and the event period, respectively.

Figure 2 shows the time courses of relative changes in oxy-Hb and deoxy-Hb averaged over all trials for all measurement channels under each condition. While the increase in oxy-Hb signals was evident before the auditory-event onset in Exp-CE, an increase was not observed in any other conditions (Exp-CNE, Cont-CE, and Cont-CNE). The event-related increases in oxy-Hb signals and slight decreases in deoxy-Hb signals were evident in response to the auditory events in both Exp-CE and Cont-CE and returned to zero baseline by the end of the trials. On the other hand, the averaged time courses in Exp-CNE and Cont-CNE did not show clear changes throughout the trial. For further analysis, we focused on oxy-Hb signals because of their large amplitude of relative signal changes compared with deoxy-Hb signals [16], [17], [21], [26], and we evaluated the increase in oxy-Hb signals during the delay period before event onset on a channel-by-channel basis to reveal the cortical regions that showed the anticipatory activation.

**Figure 3:**
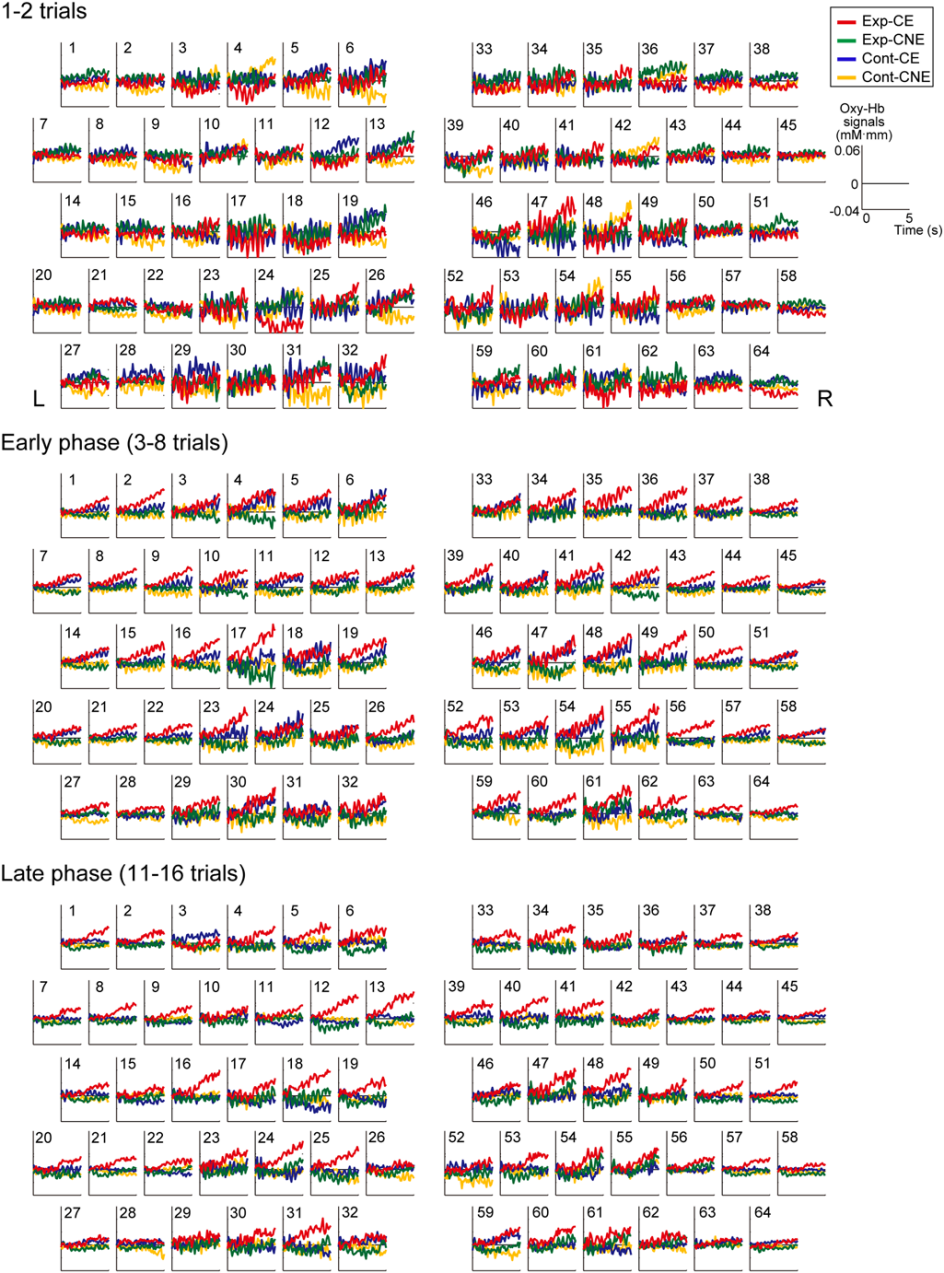
Changes in hemodynamic responses to the cue in three phases. The arrangement of panels is the same as that of the measurement channels shown in Figure 1B. The red, green, blue, and yellow lines indicate the averaged time courses of oxy-Hb signals for the first 5 s under Exp-CE, Exp-CNE, Cont-CE, and Cont-CNE, respectively.

Figure 3 shows the time courses of oxy-Hb signals for the 5 s after the cue onset (prior to the event onset) in the first two trials and in the early and late phases. We found spatiotemporal changes in cortical activation as the experimental session progressed. In the first two trials (the first trial of CE and CNE), the mean time courses of oxy-Hb for the four conditions (Exp-CE, Exp-CNE, Cont-CE, and Cont-CNE) did not show clear differences in the frontal regions. In the early phase, an increase in the oxy-Hb signal in Exp-CE was evident at some channels, especially in the bilateral frontal regions (red line). In the late phase, the bilateral temporal and temporoparietal regions as well as the frontal regions showed a distinctive increase in oxy-Hb in Exp-CE.

**Figure 4:**
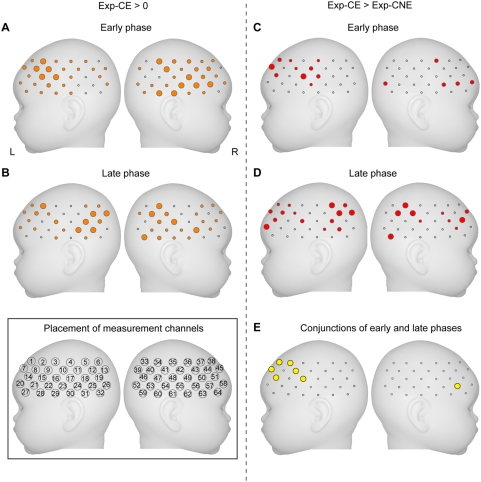
Cortical regions showing activations in response to the cue followed by the event. (A and B) The mean signal changes of Exp-CE were compared against zero baselines in the early (A) and late (B) phases. (C and D) Direct comparisons were also performed between Exp-CE and Exp-CNE in the early (C) and late (D) phases. The statistical thresholds were corrected for multiple comparisons among 64 channels using a false discovery rate with an α = 0.05. Large-, medium-, and small-filled circles indicate channels that surpassed p<0.005, 0.01, and 0.05, respectively. Small-open circles indicate channels that did not show significant activation. (E) Cortical regions showing more activation in response to the cue of Exp-CE than to the cue of Exp-CNE in both early and late phases.

We investigated the cortical regions showing anticipatory activation in the early and late phases of the experimental group by conducting statistical tests on the mean signal changes during the delay period (4.0 s–5.0 s after the cue onset) for each measurement channel (one-sample *t* test). In the early phase, the bilateral frontal and the right temporal and temporoparietal regions showed significant activations against zero baseline in response to the cue followed by the auditory events (Exp-CE, Figure 4A). The measurement channels that showed the most significant activation in each region in response to the cue of Exp-CE were Ch34 in the right temporoparietal region [*t*(25) = 3.7, p<0.0005], Ch61 in the right temporal region [*t*(24) = 3.7, p<0.0006], Ch56 in the right frontal region [*t*(27) = 3.1, p<0.002], and Ch9 in the left frontal region [*t*(27) = 3.0, p<0.003]. In contrast, there was no measurement channel in which activation in response to the cue followed by no event (Exp-CNE) surpassed the statistical threshold. In the late phase, the bilateral temporal, temporoparietal, and frontal regions exhibited significant activations in response to the cue of Exp-CE (Figure 4B). The measurement channels that showed the most significant activation in each region in response to the cue of Exp-CE were Ch24 in the left temporal region [*t*(24) = 3.8, p<0.0004], Ch13 in the left temporoparietal region [*t*(26) = 3.6, p<0.0008], Ch34 in the right temporoparietal region [*t*(25) = 3.3, p<0.002], and Ch56 in the right frontal region [*t*(27) = 3.6, p<0.0006]. There was no measurement channel in which activation in response to the cue of Exp-CNE surpassed statistical threshold in the late phase or in the early phase.

To identify the cortical regions showing an increase in activation in response to the cue of Exp-CE compared with the cue of Exp-CNE, the mean signal differences between conditions were directly compared in each phase by using paired *t* tests (Figures 4C and D). The comparisons (Exp-CE>Exp-CNE) in the early phase revealed significant differences in the bilateral frontal regions. The measurement channels of the left Ch16 [*t*(27) = 3.1, p<0.003] showed the most significant difference. In contrast, the same comparisons in the late phase revealed highly significant differences in the bilateral temporoparietal regions [left Ch12, *t*(26) = 3.8, p<0.0004; right Ch41, *t*(24) = 3.3, p<0.002]. In addition, the bilateral frontal regions also showed significant differences [left Ch20, *t*(27) = 2.9, p<0.004; right Ch51, *t*(27) = 3.2, p<0.002]. The conjunction analysis of these comparison tests revealed that the bilateral frontal regions showed significant differences in both early and late phases (Figure 4E). In the reversed comparison (Exp-CNE>Exp-CE), no significant difference was observed in any of the regions under both phases. To evaluate changes in activation over the phases, we compared the mean signal changes of Exp-CE between the early and late phases, and we also compared the mean signal differences between conditions (Exp-CE–Exp-CNE) in the early phase with those in the late phase. However, no significant difference was observed between phases.

We further examined cortical activation in the control group by using the same statistical tests used with the experimental group. When the cue was neutral, no significant activation during the delay period was observed in either condition (Cont-CE and Cont-CNE) during either phase, and there was no significant difference between conditions.

**Figure 5:**
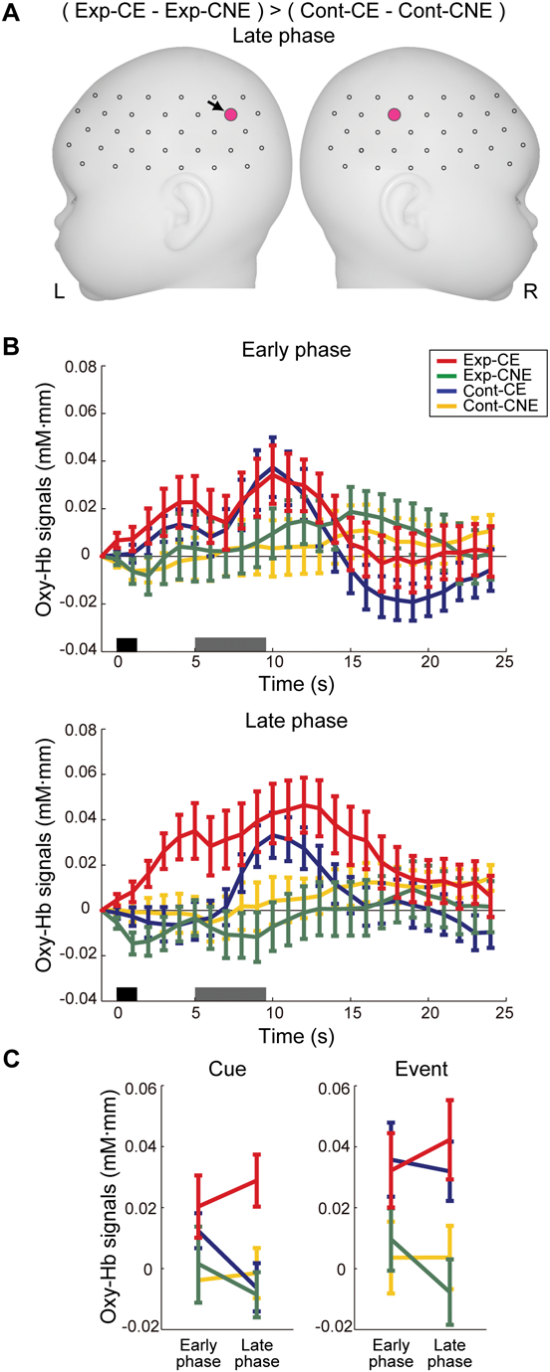
Cortical regions showing increase in oxy-Hb signals before the onset of the event. (A) Cortical regions showing specific activations in response to the cue of Exp-CE. The mean signal differences between Exp-CE and Exp-CNE were compared with the differences between Cont-CE and Cont-CNE. Pink-filled circles indicate channels that surpassed p<0.003. (B) Changes in hemodynamic responses between phases under each condition in the left temporoparietal channel (Ch12, black arrowed in Figure 5A). Error bars indicate standard errors among the infants. The dark gray bars and light gray bars indicate the cue period and the event period, respectively. (C) Changes in the magnitude of hemodynamic responses to the cue and the event between phases in Ch12. These values for the cue and the event were derived from the calculation of the mean signal changes of oxy-Hb averaged in a time window from 4.0 to 5.0 s and from 9.5 to 11.5 s after the cue onset, respectively. Error bars indicate standard errors among the infants.

To examine the difference in the cue effect between the groups, the mean signal differences between conditions in the experimental group were directly compared with those in the control group for each channel (two-sample *t* test). While no significant group difference was observed in the early phase, the bilateral temporoparietal regions showed significantly greater signal differences between Exp-CE and Exp-CNE compared with the differences between Cont-CE and Cont-CNE in the late phase (Figure 5A; left Ch12, *t*(53) = 3.1, p<0.002; right Ch41, *t*(50) = 3.1, p<0.002). Figure 5B displays the time courses of relative changes in oxy-Hb in the temporoparietal region (Ch12) under each phase. The distinctive increase in oxy-Hb signals in Exp-CE during the delay period was evident in the late phase compared with the early phase. In fact, the increase in oxy-Hb signals in Exp-CE during the delay period was augmented in the late phase compared with the early phase (Figure 5C).

We further examined the activated regions in response to the auditory events under each phase in Exp-CE and Cont-CE. The bilateral temporal, temporoparietal, and frontal regions showed significant activation against zero baseline in the early and late phases of both groups, and no significant difference in cortical activation was observed between phases and between groups (see Figure S1). The cortical regions that were activated in response to the cue of Exp-CE also showed activation in response to the auditory event. In Ch12, which showed the greatest difference in signal changes during the delay period in the late phase, the signal changes in response to the auditory event in Exp-CE was greater than those in Cont-CE, but the difference between them did not surpass the statistical threshold (Figure 5C).

## Discussion

In the present study, we found that the association cortices of sleeping infants showed anticipatory activation, which was induced by a cue that was followed by an auditory event. Such activation was not induced by a cue that was not followed by any event or by a neutral cue that was followed by the event or no-event in equal proportions. These results indicate that infants can covertly learn to associate the cue with the event based on their temporal relations during sleep. Furthermore, cortical activation only in response to the cue predicting the event suggests that this activation may reflect an implicit mechanism for anticipation of the coming of the event.

The bilateral temporoparietal regions showed prominent activation in response to the cue predicting the event only in the late phase of the experimental group. The temporoparietal region is thought to be the auditory association area, which is involved in diverse types of sound processing in adults [44]. This region also showed an increase in activation prior to the onset of the auditory event when the preceding cue indicated the coming of the event [11]. A previous study with sleeping infants reported that the right temporoparietal region is related to the processing of pitch information in speech sounds [20]. Taken together, a possible explanation for the changes in the temporoparietal activation in the present study is that learning progressed so that detection of the cue implicitly facilitated a specific mechanism of this region that processes the auditory event.

The frontal region of the experimental group showed activation in response to the cue predicting the event in both early and late phases. This result suggests that the frontal region may be involved in more general aspects of learning at an earlier stage and that the functional roles for the temporoparietal and frontal regions may differ. Previous studies with adults reported that the prefrontal cortex is involved in directing attention to the predictive future event, as well as in modulation of the activation of sensory areas before and/or after the event onset [6]–[8], [10], [11]. Although the infants in the present study could not show overt behavioral responses, the anticipatory activation of the prefrontal cortex suggests that infants may covertly attend to future events.

The present study inferred early functioning of the prefrontal cortex. A previous study on auditory habituation and dishabituation also revealed that the prefrontal cortex of infants shows activation related to novelty detection [17]. Recent studies of the anatomical development of cortex also support early functioning of the prefrontal cortex. The pyramidal cells in Layers 3 and 5 show similar, rapid dendritic growth until a few months after birth, and therefore, there appears to be sufficient structural maturity in the prefrontal cortex to support information processing in infants [45]–[47]. In addition, recent research using diffusion tensor imaging and tractography revealed that the white-matter fiber bundles connecting the temporal and prefrontal cortices are already in place in early infancy [48]. The coactivation of the frontal and temporoparietal regions in the late phase of the present study may reflect interactions between these regions.

In human adults, the results of sleep learning are inconclusive [49]. However, the present study demonstrated that infants can learn temporal associations between a cue and an auditory event during sleep. ERP studies also reported that mismatch negativity (MMN) can be observed across all sleep stages as well as in awake infants, unlike in adults [50], [51]. Moreover, previous studies on infants showed that habituation learning of auditory stimuli occurs in the sleep state [17] and infants can assimilate auditory information during sleep [52]. These findings imply that sleep in infants may serve different functional processes as compared with that in adults [53]. It should be noted that animal studies found that associative learning as well as habituation learning can occur in sleeping rats [54]–[56]. It is open to future study whether infant learning during sleep is based on the same mechanism as that while awake. The question also remains as to whether an association that has been formed during sleep is retained during the subsequent awake period.

Behavioral studies have reported that infants are able to make associations between environmental events and show anticipatory responses by a few months of age [2]–[5]. The present study provided the first evidence that 3-month-old infants can implicitly associate temporally distant events and increase cortical activation in advance of the event only when the preceding cue indicates the coming of it. Our findings imply that the anticipatory behaviors observed in infants may be attributable to cortical processes that involve the association cortices.

## Supporting Information

Figure S1 Cortical regions showing activation in response to the auditory event against zero baseline in the early and late phases of both groups. Large-, medium-, and small-filled circles indicate channels that surpassed p<0.005, 0.01, and 0.05, respectively. Small-open circles indicate channels that did not show significant activation.
(1.58 MB TIF)
