# Supplementary figures and images for: Anticipatory Cortical Activation Precedes Auditory Events in Sleeping Infants
Source: PLoS One. 2008 Dec 10;3(12):e3912. doi: 10.1371/journal.pone.0003912 (PMC2588543; doi:10.1371/journal.pone.0003912)

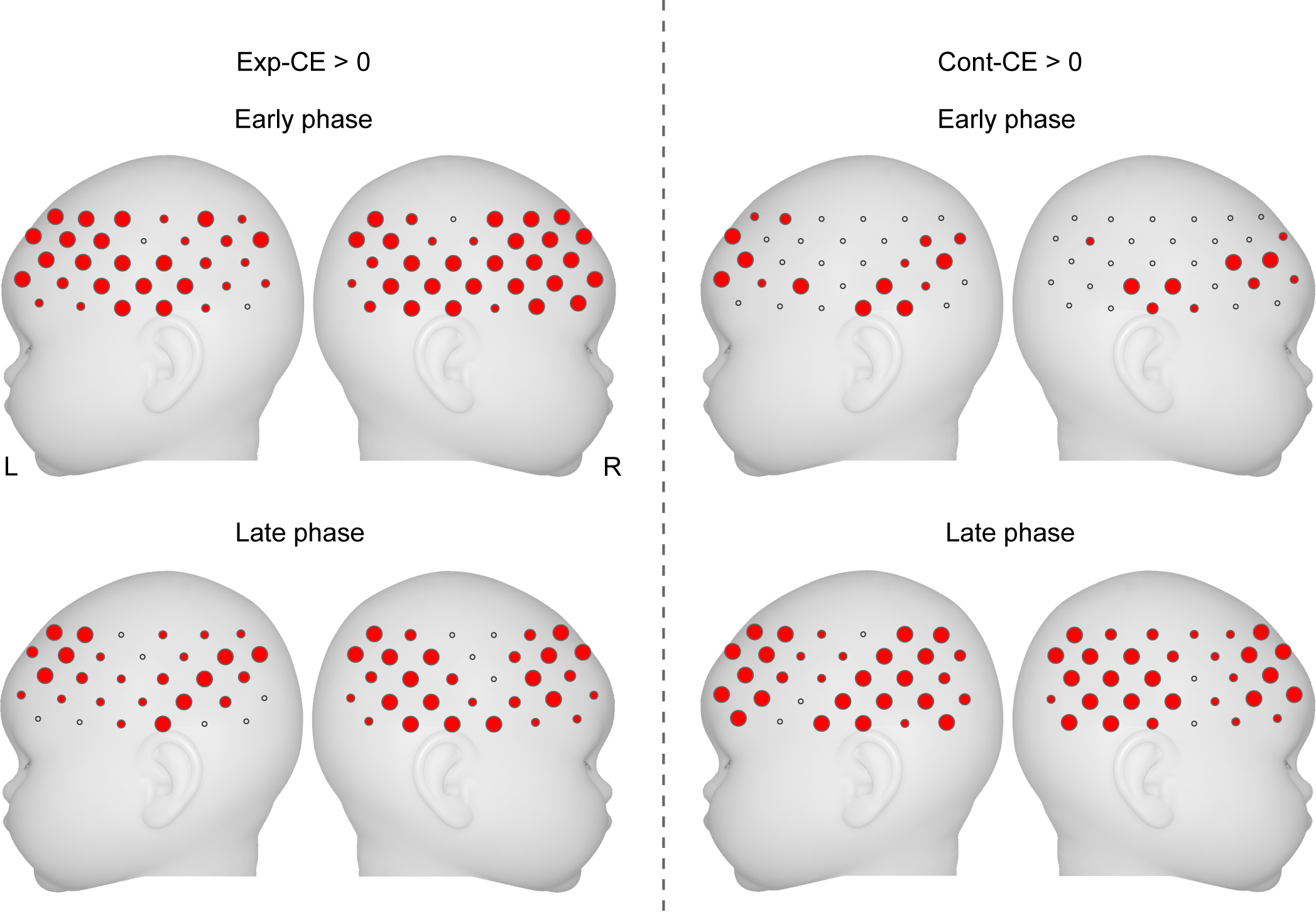

Supplement: Figure S1 — Cortical regions showing activation in response to the auditory event against zero baseline in the early and late phases of both groups. Large-, medium-, and small-filled circles indicate channels that surpassed p<0.005, 0.01, and 0.05, respectively. Small-open circles indicate channels that did not show significant activation. (1.58 MB TIF) [file pone.0003912.s001.tif]
